# Application of molecular detection, phylogenetic analysis, and risk factor evaluation for combating *Anaplasma* infection in small-scale livestock farms in Thailand

**DOI:** 10.1017/S0031182025000277

**Published:** 2025-03

**Authors:** Wissanuwat Chimnoi, Pairpailin Jhaiaun, Jumnongjit Phasuk, Domechai Kaewnoi, Tawin Inpankaew, Burin Nimsuphan, Ruttayaporn Ngasaman, Ketsarin Kamyingkird

**Affiliations:** 1 Department of Parasitology, Faculty of Veterinary Medicine, Kasetsart University, Bangkok, Thailand; 2 Faculty of Veterinary Science, Prince of Songkla University, Songkhla, Thailand

**Keywords:** *Anaplasma marginale*, bovine anaplasmosis, bovine tick-borne diseases, *Msp4*, Phylogenetic analysis

## Abstract

Anaplasmosis is a significant tick-borne disease (TBDs) caused by *Anaplasma* that affecting ruminant health and production worldwide. This study aimed to identify *Anaplasma* spp. infection using molecular as a fast diagnostic tool, perform a phylogenetic analysis and evaluate associated risk factors for combating *Anaplasma* spp. infection in small-scale livestock farms in Thailand. Total 963 blood samples from ruminants were collected from 125 farms across 4 regions of Thailand. Molecular diagnosis of *Anaplasma* spp. targeted the *msp4* gene using conventional polymerase chain reaction (PCR) was performed and reported to the farmers within 14 days. Positive PCR products were purified, sequenced, and analysed the phylogenetic. Associated risk factor evaluations were conducted using R software. The overall prevalence of *Anaplasma* spp. infection in ruminants was 26.90%. The highest prevalence was observed in bullfighting cattle (47.06%), followed by beef cattle (35.75%), dairy cattle (21.73%), and goats (6.67%), with no infection in buffalo. Regionally, the Northern region had the highest prevalence (49.01%), followed by the Southern (25.88%), Central (22.01%), and Northeastern (13.81%) regions. *Anaplasma* spp. was commonly detected in Phrae, Chiang Rai, and Tak provinces. Sequencing confirmed *A. marginale* 99.64% to 99.76% identity to sequences in GenBank. Risk factors associated with *A. marginale* infection were history of TBDs on farm, animal illnesses, responsible person for treatment, and improper faeces removal practices. This study revealed a moderate to high *Anaplasma* infection across four regions. These findings underscore the need for enhanced tick control measures on farms, should be strictly implemented and promoted to reduce disease prevalence.

## Graphical abstract

**Figure:**
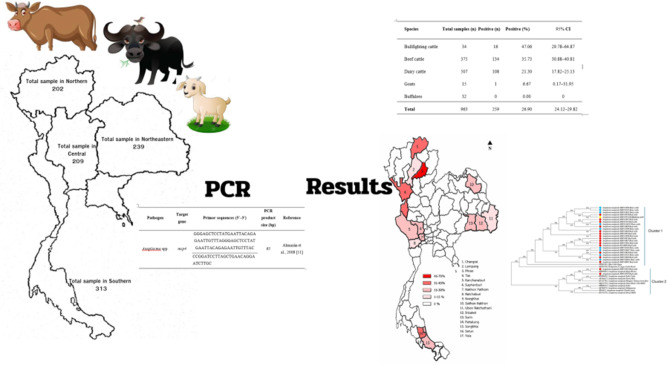

## Introduction

*Anaplasma* is an alpha-proteobacterium in the order Rickettsiales, family Anaplasmataceae, genus *Anaplasma*, which causes a tick-borne disease (TBD) known as anaplasmosis in cattle, sheep, goats, buffalo, and wild ruminants (Atif, 2016). Anaplasmosis predominantly occurs in tropical and subtropical regions worldwide (WOAH, 2024). Clinical anaplasmosis is mostly caused by infection with *Anaplasma marginale*. It is transmitted through the bites of ticks, tabanid flies, or the use of blood-contaminated instruments. After infection, *Anaplasma* invades and destroys red blood cells and the spleen, causing infected animals to become anaemic, weak, and lethargic; lose their appetite; and develop a fever (WOAH Terrestrial Manual, 2024). Clinical symptoms include pale mucous membranes, which may appear yellow. The packed cell volume of severely infected animals can become extremely low, making them prone to death. Abortion has also been associated with anaplasmosis (Capucille, 2008).

The incidence of anaplasmosis in Thailand has increased over the past decade, particularly in cattle. In a polymerase chain reaction (PCR) analysis targeting the *msp4* gene, 10.30% of cattle in the North and Northeast tested positive for *A. marginale* (Junsiri *et al*., 2002). From 2016 to 2021, the overall infection rate of *Anaplasma* spp. in cattle from 18 provinces was 46.1%, with *A. marginale* being the most common species identified (65%) (Arnuphapprasert *et al*., 2023). In 2023, 17.6% and 20.8% of cattle in the Northeast were positive for *Anaplasma* spp. by microscopic examination and DNA amplification, respectively. The prevalence in dairy cattle (23%) was higher than in beef cattle (16%) (Seerintra *et al*., 2023).

Anaplasmosis has also been reported in other ruminants in Thailand, such as water buffalo and goats. The prevalence of the family Anaplasmataceae in buffalo ranged from 6.0% to 67.6%,with an average of 41% (Nguyen *et al*., 2020). In goats from Chonburi province, 24.7% tested positive for *A. marginale* infection (Chankong *et al*., 2021). In the South, the overall rate of *A. ovis* infection was 1.5% (Udonsom *et al*., 2022). The highest prevalence of anaplasmosis was observed during heavy tick and fly seasons or when animals moved to endemic areas. Among cattle in the Chao Phraya River basin of Thailand, the overall prevalence of *A. marginale* was 14.9% during the dry season and 23.2% during the rainy season (Saetiew *et al*., 2020). Several risk factors associated with *Anaplasma* infection in livestock of Thailand including climatic condition which influences increasing the tick vector (Saetiew *et al*., 2020), host (age, gender, and breed) and environments such as ecosystem, farm management and herd size (Seerintra *et al*., 2023). Moreover, increasing the movements and exchange of livestock between the border of the country also increased the chances of blood parasite infection (Singasa *et al*., 2020). In Malaysia, risk factors significantly associated (P<0.05) with the detection of *A. marginale* in cattle were breeds, herd owner, management system, presence of ticks and frequency of de-ticking (Ola-Fadunsin *et al*., 2018).

Previous studies provided better understanding of *Anaplasma* infections however, application of molecular diagnostic tools have never been applied as routine diagnostic tools to confirm infected animals for treatment and control of this anaplasmosis. This study aimed to investigate the types of *Anaplasma* spp. using molecular as a fast diagnostic tool, perform phylogenetic analysis, and evaluate the risk factors associated with *Anaplasma* spp. infection for combating anaplasmosis in small-scale livestock farms across four regions of Thailand. The findings were communicated to farmers, and they were educated on methods to control animal anaplasmosis by reducing the identified risk factors.

## Materials and methods

### Study area and sample collection

This study was part of the integration of diagnostic services for vector-borne parasitic diseases and prevention of insect vector projects (FF(KU) 32.67). Five provinces per region of Thailand were selected for the project announcement. Official letters and phone calls via the provincial offices of the Department of Livestock Development and some national university units were made. The diagnostic service was publicly available from June 2023 to March 2024. Small-scale livestock farmers who wished to participate in this service could register through one of three methods: telephone call, provincial Department of Livestock Development offices, or an electronic platform. Samples collected from livestock followed the animal use protocol, which was reviewed and approved by the Kasetsart University Institutional Animal Care and Use Committee (approval number ACKU65-VET-095). Data on livestock management, animal husbandry, hygiene and sanitation, and livestock illness history related to vector-borne parasitic diseases were collected through interviews using questionnaires and/or electronic platforms (Google forms). Informed consent was obtained from farmers before interviews and sample collection from their animals.

### DNA extraction

In total, 200µL of whole blood samples from animals in EDTA tubes were used for DNA extraction with a blood genomic DNA extraction kit (Macherey-Nagel, Düren, Germany) following the manufacturer’s instructions. The concentration and quality of the genomic DNA were measured using an Eppendorf BioSpectrophotometer (Eppendorf AG, Hamburg, Germany) at 260 and 280nm.

### *Detection of* Anaplasma *spp*.

Conventional PCR targeting the *msp4* gene was performed using two pairs of primers (Table 1), as previously described (Almazán *et al*., 2008). The PCR master mix was prepared with 5 μL of Taq 2X master mix (New England Biolabs, Ipswich, MA, USA), 0.2 μL of each primer (20 pmol), 3.6 μL of nuclease-free water, and 1 μL of gDNA template. Thermocycler conditions included initial denaturation at 95°C for 2 minutes, followed by 40 cycles of denaturation at 95°C for 2 minutes, annealing at 60°C for 30 seconds, and extension at 72°C for 1 minute. The final extension was performed at 72°C for 3 minutes, and the samples were held at 4°C in an Eppendorf Thermocycler (Eppendorf AG). The PCR products were separated by electrophoresis on a 1.5% agarose gel at 100 V and 400 mA for 30 minutes. The gel was then visualised under UV light using a gel documentation system.

**Table 1.** Primers used for detection of *Anaplasma* spp. In this study

Pathogen	Target gene	Primer sequences (5′ − 3′)	PCR product size (bp)	Reference
*Anaplasma* spp.	*msp4*	GGGAGCTCCTATGAATTACAGA GAATTGTTTAGGGAGCTCCTAT GAATTACAGAGAATTGTTTAC	65	Almazán *et al*., 2008
		CCGGATCCTTAGCTGA ACAGGAATCTTGC		

### Sequencing analysis

Positive PCR products of *Anaplasma* spp. were selected and excised for gel extraction and purification using a gel extraction kit (Macherey-Nagel). The quantity and quality of the purified PCR products were measured using a spectrophotometer, as described above. The purified PCR products were sent for nucleotide sequencing using the Sanger method. Nucleotide sequences were analysed using Basic Local Alignment Search Tool (BLAST) on the National Center for Biotechnology Information (NCBI) platform (https://blast.ncbi.nlm.nih.gov). Multiple alignments were performed using Clustal W, and phylogenetic trees were constructed using MEGA (version 11) software. The evolutionary history was inferred using the maximum likelihood method and the Tamura-Nei model (Tamura and Nei, 1993). Evolutionary analyses were conducted in MEGA (version 11) (Tamura *et al*., 2021).

### Statistical analysis

Statistical analysis was performed using RStudio software (version 4.3.2). A t-test was used for comparisons of the *Anaplasma* prevalence among the group of animals. Chi-square test was conducted to analyse the association of factors in *anaplasma*-infected animals. Probability (*P*) values were calculated, with statistical significance defined as *P* < 0.05 at a 95% confidence interval.

## Results

In this study, 963 blood samples were collected from 125 farms across 17 provinces. The samples were obtained from five species of ruminants, including dairy cattle (n = 507), beef cattle (n = 375), bullfighting cattle (n = 34), buffaloes (n = 32), and goats (n = 15) from four regions of Thailand (Northern, Northeastern, Central, and Southern). In the Northern region, 202 samples were collected from Chiang Rai (n = 70), Lampang (n = 26), Phrae (n = 87), and Tak (n = 19) provinces. A total of 239 samples in the Northeastern region were from Nong Khai (n = 49), Surin (n = 50), Si Sa Ket (n = 50), Sakon Nakhon (n = 56), and Ubon Ratchathani (n = 34). In the Central region, 209 samples were collected from Nakhon Pathom (n = 57), Ratchaburi (n = 51), Kanchanaburi (n = 52), and Suphan Buri (n = 49). In the Southern region, 313 samples were collected from Songkhla (n = 259), Phatthalung (n = 50), Yala (n = 3), and Satun (n = 1). Molecular detection revealed an overall *A. marginale* infection rate of 26.90% (259/963). The highest prevalence was detected in bullfighting cattle (47.06%, 16/34), followed by beef cattle (35.73%, 134/375), dairy cattle (21.30%, 108/507), and goats (6.67%, 1/15), while no positive samples were detected in buffaloes (Table 2). By region, the highest prevalence of *Anaplasma* spp. was observed in the Northern region (49.01%, 99/202), followed by the Southern (25.88%, 81/313), Central (22.01%, 46/209), and Northeastern (13.81%, 33/239) regions, respectively (Table 3).

**Table 2.** Molecular detection of *Anaplasma* spp. In ruminants of Thailand

Species	Total samples (n)	Positive (n)	Positive (%)	95% CI
Bullfighting cattle	34	16	47.06	29.78 − 64.87
Beef cattle	375	134	35.73	30.88 − 40.81
Dairy cattle	507	108	21.30	17.82 − 25.13
Goats	15	1	6.67	0.17 − 31.95
Buffaloes	32	0	0.00	0
Total	963	259	26.90	24.12 − 29.82

**Table 3.** Molecular detection of *Anaplasma* spp. In each region of Thailand

Region	Total samples (n)	Positive (n)	Positive (%)	95% CI
Northern	202	99	49.01	41.93 − 56.12
Southern	313	81	25.88	21.12 − 31.10
Central	209	46	22.01	16.59 − 28.24
Northeastern	239	33	13.81	9.70 − 18.84
Total	963	259	26.90	24.12 − 29.82

**Figure 1.**
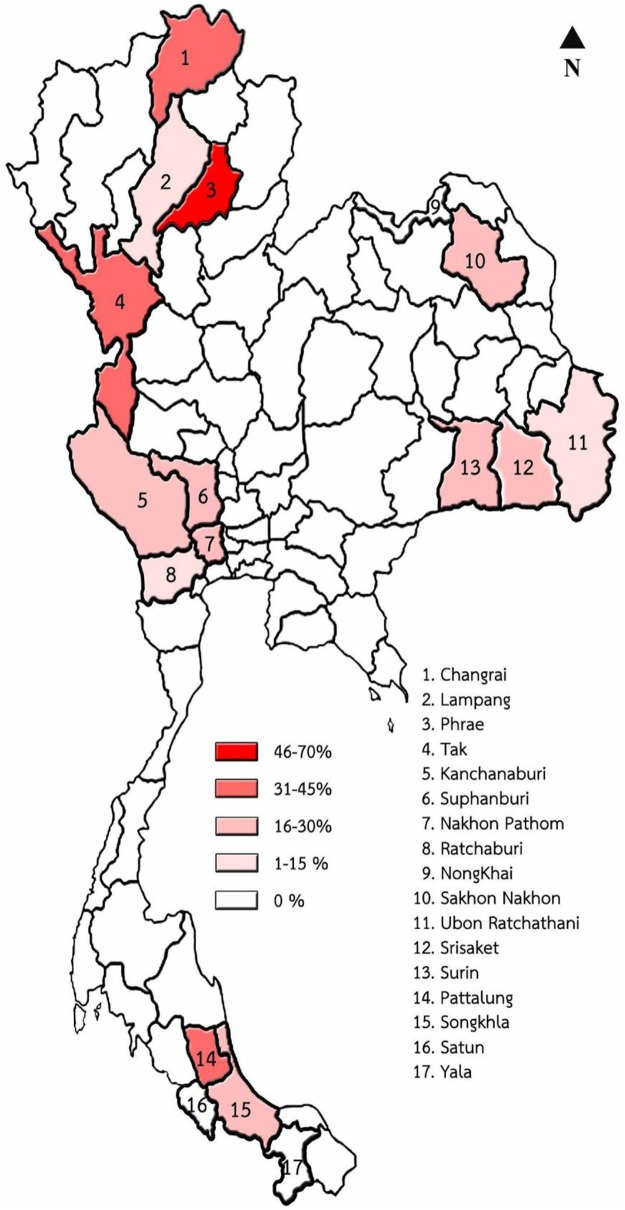
Map of positive results in each province of Thailand.

In a high-endemic area (Northern region), the province with the highest detection rate of *Anaplasma* spp. was Phrae at 66.67%. Chiang Rai and Tak provinces showed similar results, with detection rates of 42.86% and 42.11%, respectively. Samples from Lampang showed a lower positivity rate of 11.54%. In the Southern region, Phatthalung and Songkhla provinces had moderate detection rates of *Anaplasma* spp., at 40.00% and 23.55%, respectively. No positive samples were detected from the small number of blood samples collected in Satun and Yala provinces. In the Central region, Suphan Buri, Kanchanaburi, and Nakhon Pathom provinces showed positive results of 28.57%, 26.92%, and 24.56%, respectively. By contrast, Ratchaburi had a much lower positivity rate of 7.84%. In the Northeastern region, detection rates were 20.00% in Surin, 19.64% in Sakon Nakhon, 18.00% in Si Sa Ket, and 8.82% in Ubon Ratchathani, with no positive samples detected in Nong Khai (Table 4, Figure 1).

**Table 4.** Molecular detection of *Anaplasma* spp. In selected provinces of each region of Thailand

Region	Province	Total samples (n)	Positive (n)	Positive (%)	95% CI
Northern	Phrae	87	58	66.67	55.75 − 76.42
	ChiangRai	70	30	42.86	31.09 − 55.25
	Tak	19	8	42.11	20.25 − 66.55
	Lampang	26	3	11.54	2.45 − 30.15
Southern	Pattalung	50	20	40.00	26.41 − 54.82
	Songkhla	259	61	23.55	18.52 − 29.20
	Satun	1	0	0.00	0
	Yala	3	0	0.00	0
Central	Suphanburi	49	14	28.57	16.58 − 43.26
	Kanchanaburi	52	14	26.92	15.57 − 41.02
	Nakhon Pathom	57	14	24.56	14.13 − 37.76
	Ratchaburi	51	4	7.84	2.18 − 1.88
Northeastern	Surin	50	10	20.00	10.03 − 33.72
	Sakon Nakhon	56	11	19.64	10.23 − 32.43
	Srisaket	50	9	18.00	8.58 − 31.44
	Ubon Ratchathani	34	3	8.82	1.86 − 23.68
	Nong Khai	49	0	0.00	0
Total		963	259	26.90	24.12 − 29.82

**Figure 2.**
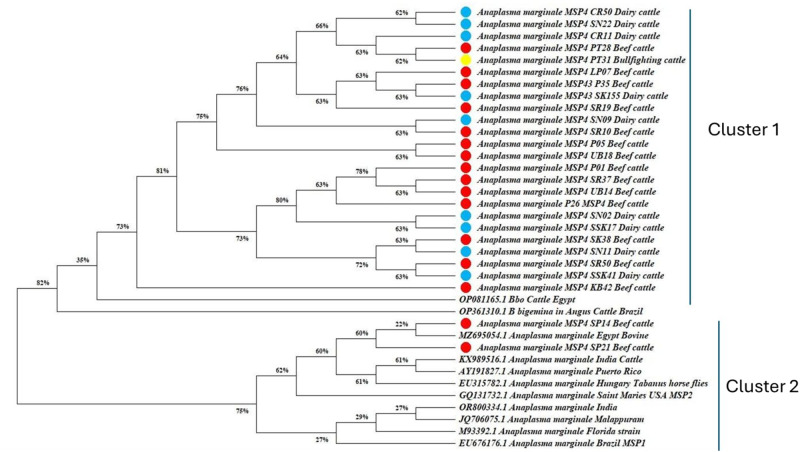
Phylogenetic tree of *Anaplasma marginale*. msp4 gene Thai isolates. The tree with the highest log likelihood (−11,456.83) is shown. Initial tree(s) for the heuristic search were generated automatically by applying the neighbor-join and bionj algorithms to a matrix of pairwise distances estimated using the tamura-nei model, and the topology with the highest log likelihood value was selected. The proportion of sites with at least one unambiguous base present in at least one sequence for each descendent clade is shown next to each internal node in the tree.

Phylogenetic analysis revealed that *A. marginale* in Thailand formed two clusters with six clades. The robust nodes of clusters 1 and 2 were 82% and 75%, respectively. Sequence alignment of positive samples from beef cattle, dairy cattle, and bullfighting cattle showed similarity to three deposited sequences in GenBank (accession numbers OP081165.1, OP361310.1, MZ695054.1) from Egypt and Brazil (Figure 2).

Questionnaires were used to collect data on risk factors and farm management practices, with 123 of 125 farms (97.62%) participating in the interviews. The results identified significant factors associated with infection, including a history of TBDs, a history of illness, the individual responsible for treating diseased animals, and the removal of faeces from farms (*P* ≤ 0.05). Farms with a history of TBDs had a significantly higher prevalence of *A. marginale* infection (87.58%) than farms without such a history (56.45%). Similarly, a history of illness on the farm was significantly associated with *A. marginale* infection (72.86%) compared with the absence of a history of illness (42.31%). Symptoms significantly associated with infection included pale mucous membranes (*P* = 0.021), weight loss (*P* = 0.005), and excessive lachrymation (*P* = 0.005). On farms where farmers themselves treated sick animals, *A. marginale* infection was higher (88.00%) than on farms where a veterinarian was responsible (57.81%) (*P* = 0.007). Interestingly, faeces removal was also identified as a significant factor: farms that removed faeces showed a higher prevalence of infection (67.35%) than farms that did not (35.71%) (*P*=0.022). This might because of limitation of sample size in non-faeces removal farm (N=14). Moreover, from observation farms that removed the faeces did improper cleaning method. The farmer kept the faeces nearby the stable, have no specific area for make it dry as fertilizer. Although the frequency of faeces removal did not show a statistically significant difference in infection rates (*P*=0.053), farms that removed faeces monthly had the highest infection rate (90%), followed by weekly (80.00%), twice weekly (72.73%), and daily (62.12%) (Table 5).

**Table 5.** The factors associated with *Anaplasma marginale* infection in small-scale livestock farms in Thailand

Risk factors	Answers	No. of farms	%	Positive farms	%	Chi-square	df	P-value
History of tick-borne diseases	No	62	68.89	35	56.45	1.064	1	0.044
	Yes	28	6	22	78.57			
History of Surra	No	60	75.95	37	31.65	4.544	2	0.103
	Yes	13	16.46	12	7.59			
	Don’t know	6	7.59	4	3.8			
History of illness	No	52	42.62	22	42.31	11.586	1	0.001
	Yes	70	57.38	51	72.86			
	Pale membrane	28	22.95%	22	78.57	5.308	1	0.021
	Intermittent Fever	29	23.77%	23	79.31	6.003	1	0.014
	Weight loss	43	35.25%	33	76.74	7.899	1	0.005
	Hematuria	18	14.75%	13	72.22	1.348	1	0.246
	Anorexia	50	40.98%	35	70	3.642	1	0.056
	Lachrymal	22	18.03%	18	81.82	5.397	1	0.02
Report to local vets on animal illness	Report	88	76.52%	51	57.95	3.468	1	0.063
	Not report	27	23.48%	21	77.78			
Quarantine of the sick animals	Quarantine	85	75.22%	55	64.71	0.002	1	0.968
	Not Quarantine	28	24.78%	18	64.29			
Treatment of sick animals	Treated	108	97.30%	71	65.74	0.001	1	0.973
	Not treated	3	2.70%	2	66.67			
Who treated the sick animals?	Farmer	25	28.09%	22	88	7.331	1	0.007
	Veterinarian	64	71.91%	37	57.81			
History of animal death in the farm	Yes	13	13.83%	10	10.64%	0.669	1	0.413
	No	81	86.17%	53	56.38%			
Type of farming	Grazing	6	5.04%	4	66.67	0.173	2	0.917
	Semi-grazing & pen	41	34.45%	24	58.54			
	Stable	72	60.50%	44	61.11			
Type of Feeding	Concentrates & Roughage	30	25.21%	18	60	4.155	3	0.245
	Cutting grass	48	40.34%	28	58.33			
	Cutting grass, concentrates & roughage	10	8.40%	9	90			
	Grazing & cutting grass	31	26.05%	17	54.84			
Presence of ectoparasites	No	10	8.55%	4	40	2.143	1	0.143
	Yes	107	91.45%	68	63.55			
	Stomoxys	87	81.31%	57	65.52	2.269	1	0.132
	Haematobia	51	47.66%	30	58.82	0.282	1	0.596
	Tabanus	72	67.29%	49	68.06	3.359	1	0.067
	Ticks	44	41.12%	27	61.36	0.001	1	0.976
	Louse	14	13.08%	7	50	0.895	1	0.344
	Midges	18	16.82%	10	55.56	0.322	1	0.571
Prevention of ectoparasites	No	16	13.56%	8	50	0.945	1	0.331
	Yes	102	86.44%	64	62.75			
How often do they prevent ectoparasites?	Irregularly	52	47.27%	32	61.54	1.362	3	0.715
	Once a year	23	20.91%	13	56.52			
	Others	10	9.09%	6	60			
	Twice a year	25	22.73%	18	72			
Stool removing	No	14	12.5	5	35.71	5.282	1	0.022
	Yes	98	87.5	66	67.35			
	Daily	66	59.46%	41	62.12			
	Once a week	10	9.01%	8	80			
	Twice a week	11	9.91%	8	72.73	9.367	4	0.053
	Never	14	12.61%	5	35.71			
	Others	10	9.01%	9	90			
Position of stool trash	Near to the animal barn	77	74.04%	46	59.74	0.405	1	0.524
	Far from animal barn	27	25.96%	18	66.67			
Observation of garbage management	Clean and tidy	86	86.87%	58	67.44	0.177	1	0.674
	Dirty and full of trash	13	13.13%	8	61.54			
Water management	Presence of waste pond	20	18.18%	14	70	3.888	3	0.274
	Presence of waste drainage	32	29.09%	23	71.88			
	No waste drain system	57	51.82%	31	54.39			
	Drainage & Pond	1	0.91%	1	100			

## Discussion and conclusion

This research revealed the endemicity of *A. marginale* infection in ruminants in Thailand, with an overall prevalence of 26.90%. The infection was distributed across the country, with the highest prevalence observed in the Northern region (49.01%), particularly in Phrae province (66.67%). Most samples from this province were from beef cattle. In dairy cattle from Chiang Rai, the prevalence of *A. marginale* infection was also high (42.86%), comparable to the prevalence in beef cattle from Tak (42.11%). By contrast, the infection rate in beef cattle from Lampang was lower (11.54%). The results of this study differ from a previous study conducted in northern Thailand, which reported the prevalence of *A. marginale* infection in cattle as 85.53% in Lampang, 75.41% in Phayao, 33.57% in Lamphun, 3.50% in Chiang Mai, and 2.30% in Chiang Rai (Koonyosying *et al*., 2022). A nationwide study detecting major surface proteins revealed the highest prevalence of Anaplasmataceae family infections in the Southern region, followed by the Western, Central, Northeastern, and Northern regions, with most positive results attributed to *A. marginale* (Arnuphapprasert *et al*., 2023). From past to present, *A. marginale* has been circulating in livestock in the North, with fluctuating incidence over time. This variability may be influenced by local factors, including the presence of live animal markets and animal trading hubs in northern provinces such as Chiang Mai, Lamphun, and Phayao.

Bullfighting cattle showed a higher prevalence of *A. marginale* infection than did beef cattle, dairy cattle, and goats, with no infections detected in water buffalo. This may be related to animal movement and management practices. Bullfighting cattle, which are an indigenous breed valued for their strength, are bred and raised specifically for fighting in arenas. Before competitions, these cattle are kept near the arena for several days, during which they are housed and exercised in close proximity, often within 10m of one another, and might occupy areas previously used by other bulls. These conditions increase the likelihood of contact with vectors of blood parasites, such as ticks. Previous studies have indicated that bullfighting cattle are resistant to certain blood parasite infections, including *Theileria* spp. and *Trypanosoma evansi* (Kamyingkird *et al*., 2020; Rakwong *et al*., 2022). While microscopic detection of *Anaplasma* spp. in bullfighting cattle has shown very low positivity rates (0.10%) (Ngasaman *et al*., 2021), molecular detection in this study revealed a much higher prevalence (47.06%). This finding underscores the importance of molecular detection for blood parasite infections in bullfighting cattle before they are introduced to the arena.

According to this study, the prevalence of *A. marginale* infection in beef cattle (35.73%) was higher than in dairy cattle (21.30%). This may be because small-scale beef cattle farms typically allow animals to graze in fields rather than keeping them in stables like dairy cattle. Consequently, beef cattle have more prolonged exposure to infected vectors in the field. However, the prevalence of *A. marginale* infection in native beef cattle was consistent across all regions. This finding aligns with a previous report from the Central region, which documented a high and stable prevalence of approximately 30% in Nakhon Sawan province (Saetiew *et al*., 2020). In the Northeastern region, the prevalence was 20.8% (Seerintra *et al*., 2023). The prevalence in goats (6.67%) was lower than the 24.7% reported in goats from Chonburi province (Chankong *et al*., 2021), but the small sample size in this study (15 goats) limits meaningful comparison. Future studies should include a larger sample size for more reliable conclusions. Interestingly, this research did not detect *A. marginale* infection in buffalo from Yala, Songkhla, Lampang, and Ubon Ratchathani provinces, whereas a previous study reported a high prevalence (42.7%) in buffalo from Chachoengsao province (Nguyen *et al*., 2020)

The phylogenetic tree revealed similarities with sequences from Egypt and Brazil. Egypt is highlighted as an endemic area for *A. marginale* in asymptomatic cattle, with a prevalence of 39.6% detected by PCR assay (Metwally *et al*., 2023). Previous reports indicated a seroprevalence in cattle ranging from 18.5% to 20.0% (Parvizi *et al*., 2020; Selim *et al*., 2021). Studies using cELISA, microscopic examination, and PCR techniques reported prevalence rates of 47.40%, 47.40%, and 67.37%, respectively, in camels (Barghash and El-Naga, 2016; Parvizi *et al*., 2020). More recently, microscopic diagnosis indicated an overall seroprevalence of anaplasmosis among camels of 18.60% (Alsubki *et al*., 2022). In Brazil, anaplasmosis is considered an endemic disease in cattle, with seroprevalence rates ranging from 91.25% to 97.50% and a high genetic diversity of *A. marginale* (Garcia *et al*., 2022). Among calves, blood smears showed a 48.0% infection rate with *A. marginale*, while real-time PCR detected a positivity rate of 70.2% (Pohl *et al*., 2013). In neighbouring countries, *A. marginale* was endemic among cattle in Peninsular Malaysia, with a prevalence of 72.6% (Ola-Fadunsin *et al*., 2018). In Indonesia, the prevalence of bovine anaplasmosis in Jambi Province from 2018 to 2022 was 16.66%, with a fluctuating pattern throughout the year [26].

In this study, the significant risk factors associated with *A. marginale* infection included a history of TBDs on the farm, a history of other illnesses in animals, the person responsible for treating diseased animals, faeces removal practices, and farm cleaning programmes. By contrast, a previous study identified breed and sex as significant factors associated with *A. marginale* infection (Seerintra *et al*., 2023). Among goats, risk factors for *Anaplasma* spp. infection included age, breeding practices, acaricide use, tick infestation, and contact with cattle [20]. In Pakistan, it was reported that animals with tick infestations, those cohabiting with dogs, or those where dogs carried ticks had a higher risk of *A. marginale* infection (Asif *et al*., 2022). Although this study did not observe animals cohabiting with ruminants, the small-scale farming system, where animals frequently stay outside stables, increases the likelihood of accidental tick infestations. Based on the identified risk factors, TBD pathogens may persist on farms with a history of clinical symptoms such as pale mucous membranes, intermittent fever, weight loss, and lachrymation. These symptoms are characteristic of blood parasite infections, including anaplasmosis, which destroys red blood cells, leading to anaemia and causing animals to appear weak, pale, jaundiced, and febrile (Abdullah *et al*, 2020, Ziam *et al*., 2020). Additionally, farms where disease treatment is managed by farmers, rather than veterinarians, may have an increased risk of *A. marginale* infection, possibly due to improper or irrational drug use.

Therefore, small farming systems in Thailand should focus on implementing proper faeces removal practices and establishing regular farm cleaning programmes managed by the farmers. Local veterinary authorities should provide free services for disease treatment and support. Farmers should be educated on good farm management practices and vector control measures. Additionally, disease surveillance should be conducted at the small-farm level, with data collection carried out annually across the country to prevent the recurrence of these diseases.
